# Change detection versus change localization for faces, houses, and words

**DOI:** 10.1177/03010066231191193

**Published:** 2023-08-08

**Authors:** Paulo Ventura, Alexandre Pereira, Francisco Cruz, João Delgado, Bruno Faustino, José Carlos Guerreiro

**Affiliations:** 37809 Universidade de Lisboa, Portugal; 70887 Universidade Lusófona University, Portugal; 37809 Universidade de Lisboa, Portugal; 37809 Universidade de Lisboa, Portugal; Lusófona University, HEI-Lab: Digital Human-Environment Interaction Labs, Portugal; 37809 Universidade de Lisboa, Portugal

**Keywords:** holistic processing, change detection, change localization, faces, houses, words

## Abstract

Holistic processing aids in the discrimination of visually similar objects, but it may also come with a cost. Indeed holistic processing may improve the ability to detect changes to a face while impairing the ability to locate where the changes occur. We investigated the capacity to detect the occurrence of a change versus the capacity to detect the localization of a change for faces, houses, and words. Change detection was better than change localization for faces. Change localization outperformed change detection for houses. For words, there was no difference between detection and localization. We know from previous studies that words are processed holistically. However, being an object of visual expertise processed holistically, visual words are also a linguistic entity. Previously, the word composite effect was found for phonologically consistent words but not for phonologically inconsistent words. Being an object of visual expertise for which linguistic information is important, letter position information, is also crucial. Thus, the importance of localization of letters and features may augment the capacity to localize a change in words making the detection of a change and the detection of localization of a change equivalent.

Face recognition has been viewed as an exceptional case of object recognition (Farah et al., 1998). A large body of research supports the hypothesis that the human visual system does not process a face as a collection of separable facial features but as an integrated perceptual whole (Diamond & Carey, 1986; Gauthier & Tarr, 2002; McKone et al., 2012; Richler & Gauthier, 2014).

One of the most prominent ideas is that faces are processed holistically—as unified wholes or “gestalts”—compared to other objects that are processed in a more analytic, part- or feature-based manner (Farah et al., 1998). Unlike objects that are typically identified at the category level (e.g., “dog”; Rosch et al., 1976), it is often necessary to identify faces at the level of the individual (e.g., “Bob”). But all faces consist of the same kinds of features (eyes, nose, and mouth) in the same configuration (eyes above nose and nose above mouth). Holistic processing is believed to facilitate the individuation of such visually similar objects. One common assumption is that we quickly build holistic representations to extract useful second-order information provided by the variation between the faces of different individuals (Taubert et al., 2011).

An alternative account suggests holistic processing is a fast, early grouping process that first serves to distinguish faces from other competing objects (Taubert et al., 2011). From this perspective, holistic processing is a quick initial response to the first-order information present in every face. Holistic processing has been considered the outcome of an attentional strategy that has become automatized with experience (Richler, Palmeri, & Gauthier, 2012; Richler, Wong, & Gauthier, 2011). According to this view, face parts are encoded and represented independently, and holistic processing arises from the obligatory encoding of all object parts because a strategy of attending to all parts cannot be “turned off”. A related view is that independently represented face parts are not treated independently during perceptual decision-making, which could be the cause of holistic effects (e.g., Richler, Gauthier, Wenger, & Palmeri, 2008; Richler, Tanaka, Brown, & Gauthier, 2008).

Faces and words are both stimuli that are composed of parts with high similarity (the features of faces and the letters of words) and for which most humans have a high level of expertise. Wong, Bukach, Yuen, et al. (2011), Wong, Bukach, Hsiao, et al. (2012), Chen et al. (2013), and Ventura et al. (2017) have recently shown evidence for the holistic processing of words. At the behavioral level, the expressions of holistic processing previously found for faces, that is, the inversion (e.g., Wong et al., 2010; Yin, 1969), the part–whole (e.g., Tanaka & Farah, 1993), and the composite (e.g., Young et al., 1987) effects, have also been reported in visual word recognition.

In the inversion effect, for example, visual word recognition is impaired when words are presented inverted rather than upright (e.g., Carlos et al., 2019). Evidence for the processing of configural information was found by Wong et al. (2019). They showed that fluent readers were more sensitive to differences in configural information (jittering of part/letter positions) between two simultaneously presented words in the familiar upright orientation than in the unfamiliar inverted orientation. This effect mimics that for faces, where the inversion of the stimuli leads to worse discrimination performance, especially when configural rather than featural differences are involved (Rakover, 2013). These findings suggest that holistic processing for words and faces is both related to the acquisition of expertise and runs counter to the idea that holistic and part-based are two extremes of object recognition (Farah, 1991, 1992; Farah et al., 1998; Tanaka & Farah, 1993).

In the part–whole task, it is easier to perceive a feature (e.g., a letter and eyes) when it is presented on a whole object (e.g., a word or face) than when it is presented separately, indicating facilitation of the whole on part processing (Tanaka & Farah, 1993; Tanaka & Simonyi, 2016). The common interpretation is that object parts are not represented in isolation but instead integrated as a larger chunk or unit possibly covering the whole object. The part–whole task has been implemented slightly differently in the face and word recognition literature. For faces, the effect has been typically shown in terms of better performance for parts shown in a whole face than when presented in isolation (Tanaka & Farah, 1993). A similar task for words can be found in the classic Reicher–Wheeler paradigm (Reicher, 1969; Wheeler, 1970). The word superiority effect refers to the better recognition of a target letter presented within a word than alone or a nonword (Reicher, 1969; Wheeler, 1970). This word superiority effect is regarded as the result of the interaction between whole-word lexical representations (top-down influences) and low-level bottom-up processing at the letter level (e.g., McClelland & Rumelhart, 1981; Rumelhart & McClelland, 1982).

The face composite effect shows that all parts of a face are fully processed even if the task requires a decision on a part only. The composite task has been recently adopted to examine the holistic processing of visual words in alphabetic and logographic scripts (Chen et al., 2013; Ventura et al., 2017; Wong, Bukach, Hsiao, Greenspon, Ahern, & Duan, 2012). This is a perceptual task that does not require reading: participants are asked to perform a same-different matching task on a specific visual part (e.g., the first syllable of two consecutive [disyllabic] words and not on whole strings; same-response trials: LANE—LADY and LANE—LANE; different-response trials: LANE—CONE and LANE—COZY). Two essential components of this task argue for the need for holistic word processing. First, the influence of the irrelevant part (e.g., the right half) on performance with respect to the target part (e.g., the left half), that is, a significant congruency effect: better performance when the irrelevant part is congruent in response to the one induced by the critical part (in same-response trials: e.g., LANE—LANE, as the critical and irrelevant parts are the same; in different-response trials: LANE—COZY, as both the critical and irrelevant parts induce a different response), than when it is incongruent (in same-response trials: e.g., LANE—LADY, because the critical part of the two words is the same but the irrelevant part is different; in different-response trials: LANE—CONE, as the critical part of the words is different but the irrelevant part is the same). Second, the congruency effect is modulated by alignment; that is, it is drastically diminished when the two parts of a word are misaligned (e.g., the right part is moved down relative to the left part) rather than aligned, likely because the entire percept is disrupted.

The holistic processing literature holds that holistic processing is usually beneficial for object processing, more specifically in helping in the individuation of objects made with a similarly organized set of features (e.g., faces and words; cf. the discussion above). An intriguing possibility is that whether holistic processing is useful depends on task demand. The literature does not hold that holistic processing is always beneficial. In fact, in some cases, holistic processing may come with a cost. Wilford and Wells (2010), for instance, hypothesized that holistic processing may improve the ability to detect changes to a face while impairing the ability to locate where the changes occur. Wilford and Wells (2010) employed a change-blindness paradigm in which they presented participants with faces (requiring holistic processing) and houses (requiring part-based processing) and asked whether a change had occurred. In the other task, the test object was constantly altered, and participants were asked to identify the alterations. This new condition was critical for testing Wilford and Wells’s (2010) claim that holistic processing aids in change detection but hinders change localization.

They discovered that participants could detect changes to a face better than changes to a house, but localization of where the changes were was better for a house than a face. In Wilford and Wells (2010), inversion changed the task performance difference across faces and houses. Specifically, better performance was found in detection for upright faces and localization for inverted faces, while performance was always better in localization for houses. Mathis and Kahan (2014) found similar results using Kanizsa rectangle patterns that were either arranged to form a Gestalt whole or not. Poljac et al. (2012) reached a similar conclusion using a different paradigm with biological motion figures.

The results of Wilford and Wells (2010) can be understood in terms of Hochstein and Ahissar's reversed hierarchy theory (Hochstein & Ahissar, 2002; Hochstein et al., 2015). The information on which conscious perception is based, according to this model, is extracted from the most abstract (and thus global and meaningful) stage of visual information processing, and that feedback mechanism is required to go back and extract the details from which this abstraction is made. This efficient and fast processing at an abstract level happens for objects of expertise/holistic processing and facilitates change detection. We tend not to use this abstract processing for other objects because it is not as effective.

In a previous study (Ventura et al., 2022), we examined the accuracy of change detection and change localization for African and Caucasian faces and houses. Change detection was better than change localization for Caucasian faces. For houses, change localization was easier than detection. While clear costs of holistic processing for Caucasian faces were identified, there were no differences between change detection and change localization for African faces. However, the amount of childhood exposure to people of other races was associated with change detection performance for African faces, but not change localization performance for African faces. Thus, our findings showed that the holistic processing of faces of other races may depend on early contact with those faces of other races.

Given the findings of holistic processing for faces and words that were discussed previously (inversion task, part–whole task, and composite task), the present study evaluated whether the cost of holistic processing would be generalized to words. In a recent study (Ventura et al., 2022), we took a step further and directly showed a reciprocal interference between the holistic processing of word and face stimuli. We presented words (aligned or misaligned) superimposed on faces (aligned or misaligned) and tested the interference from the unattended stimulus category on holistic processing of the attended category. Faces were processed less holistically when an aligned word (processed holistically) was superimposed, while words were processed less holistically when an aligned face (processed holistically) was superimposed. This finding evidenced a trade-off in the holistic processing of the two stimuli, suggesting that faces and word stimuli rely, at least in part, on similar holistic processing mechanisms.

Indeed, words are objects of expertise for which we use holistic processing. Recently, Ventura et al. (2020) showed that holistic processing of visual words is related to higher efficiency in visual word recognition by skilled readers. Thus, this might contribute to high performance in change detection. However, the processing of information about features/letter identities and letter positions is also fundamental to the reading process (e.g., Grainger, 2017). Despite the possibility of some noise in encoding positional information (e.g., bigrams; Grainger & van Heuven, 2003; Perea & Lupker, 2003), the information about the location of features/letters is crucial in orthographic processing. Thus, the ability to locate where the changes occur in the change localization task is increased. and might contribute to high performance in change localization.

In the present study, we adopted the change-blindness paradigm as in Wilford and Wells (2010) and compared change detection and change localization performance for faces, houses, and words. Taken together, the extant literature indicates that holistic, meaningful patterns are extracted from the visual environment rapidly and effortlessly and that the whole tends to dominate our perception in a manner that may hinder the processing of constituent features. People should easily be able to detect these holistic changes in words, since the whole, rather than the parts, will be the focus of attention. In addition, if seeing the whole impairs our ability to see the local elements, then this ease for detecting holistic changes should come with the cost of identifying the aspect of the local elements that were modified to create these changes. We might even predict that the difference between detection and localization is higher for words due to their strong cohesiveness. This cohesiveness probably reflects the need to deal effectively with many letters in rapid temporal succession and the fact that in usual reading contexts, words appear close to other words both spatially (in terms of location) and temporally (as many words are recognized within a short time window). The human brain, therefore, needs to ensure that letters belonging to the same word are grouped into the same perceptual unit rather than mixed with letters from neighboring words. Such an organization of the complex visual display of letters into representational objects may be crucial in satisfying the highly demanding visual task of word recognition and reading.

However, being an object of visual expertise processed holistically, visual words are also a linguistic entity. In Ventura et al. (2019), participants saw two sequentially presented consonant.vowel-consonant.vowel words and had to decide if the left half (first syllable) was the same or not, regardless of the right half. The word pairs were either phonologically consistent (univocal orthography to phonology mapping; e.g., TI is always /ti/ in Portuguese) or inconsistent (orthography can map into different phonological representations; e.g., CA can correspond to /ka/ or /kɐ/). The word composite effect was found for phonologically consistent words but not for phonologically inconsistent words. These findings suggest that the holistic processing of visual words is modulated by the automatic activation of lexical phonological representations. Being an object of visual expertise for which linguistic information is important, letter position information, is also crucial. Efficient visual word recognition (i.e., fast access to abstract orthographic representations at the mental lexicon, which then act as a key interface to phonological and conceptual representations) requires fast identification of letters (from a limited set) and of their position within-word (e.g., 〈GOD〉 is different than 〈COD〉, although they only differ in a minute horizontal segment of the first letter; and 〈GOD〉 is different than 〈DOG〉). This might rise performance in change localization and thus equalize performance in detection and localization tasks.

## Method

### Participants

According to More Power (Version 6.0.4; Campbell & Thompson, 2012), a sample size of 60 would be required to detect a medium-sized effect (with η_p_^2^ = .10) at α = 0.05 with a power of 0.9 for a repeated-measures analysis of variance (ANOVA) with a 3 × 2 × 2 within-subject factors: 3 (image type: Caucasian faces vs. houses vs. words) × 2 (task: change detection vs. change localization) × 2 (image orientation: upright vs. inverted). Wilford and Wells (2010) used a 2 (image type) × 2 (task) × 2 (image orientation), which could not be used to estimate effect size in this study. We invited all participants enrolled in an introductory psychology course (78) forecasting some sample attrition.

This study's protocol adhered to the guidelines of the Declaration of Helsinki, the Portuguese deontological regulation for Psychology, and was approved by the Deontological Committee of Faculdade de Psicologia of Universidade de Lisboa. All participants provided written informed consent. Participants received course credit.

### Material

**Figure 1.**
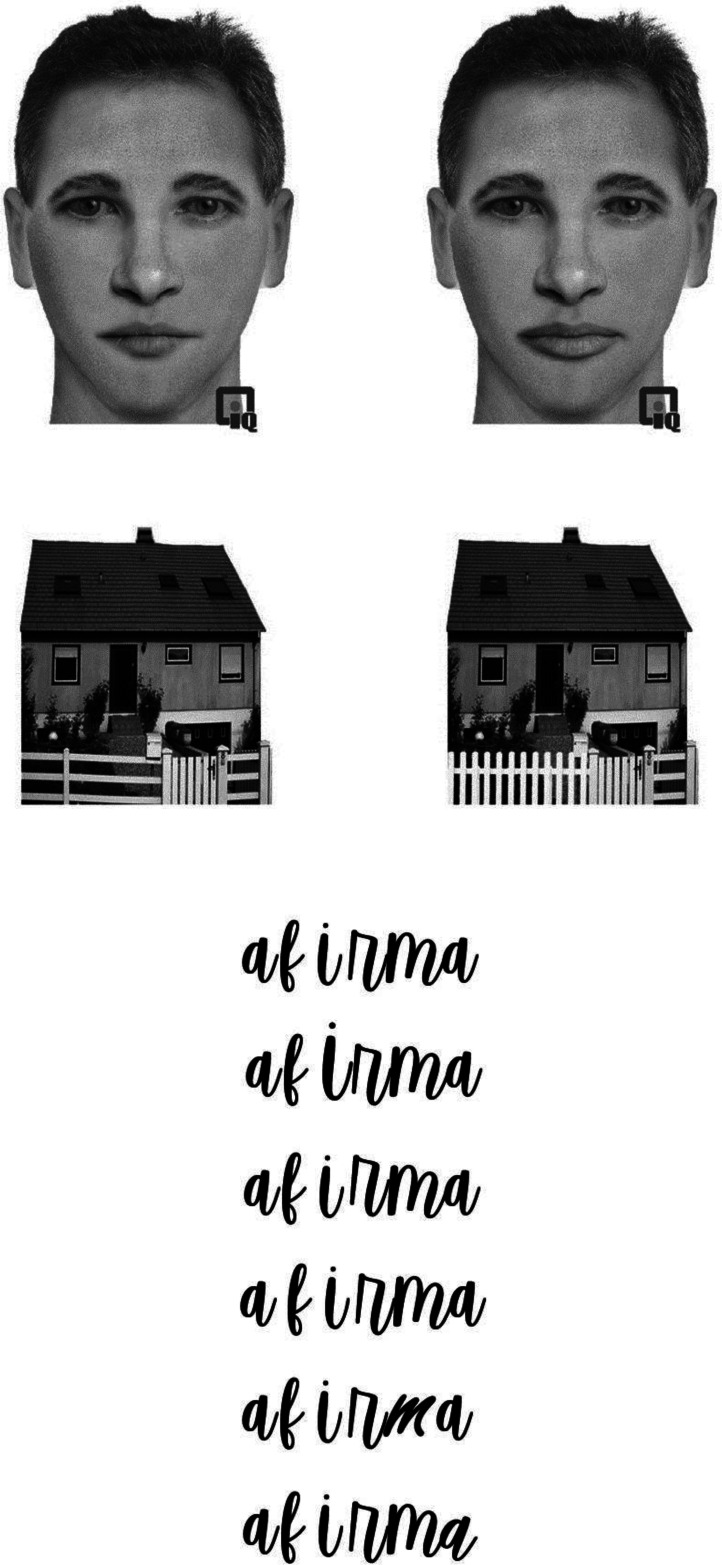
(a) Examples of the faces and houses used in the experiment. For each category, the left and right images are the original and the altered versions, respectively. In these examples, the lips of the face and the fence of the house were changed. The symbol appearing on the bottom of each face picture is a trademark of the Faces™ software (IQ Biometrix, Inc., http://www.iqbiometrix.com/products_faces_40.html) and appeared on all the face images in the experiment. (b) From top to bottom: original version, size of letter, jittering between letters/parts, space between letters, font, and rotation of a letter.

The task was similar to that of Wilford and Wells (2010). Ninety images were created for this study. These included six original Caucasian faces, six original houses, and six original words. Five variations of each face, each house, and each word were created by changing one feature at a time—for faces: hair, nose, eyes, mouth, or chin;—for houses: roof, chimney, window, door, or porch;—for words: the size of a letter (increase, decrease), font (vfree, serendipity; original font: vanilla script), rotation of a letter (clockwise and counterclockwise), vertical jittering (up and down), and spacing between adjacent letters (increase and decrease). The faces were created and manipulated utilizing Faces™ software (IQ Biometrix, Inc., http://www.iqbiometrix.com/products_faces_40.html). Houses and words were manipulated with Adobe Photoshop. See Figure 1 for examples of materials.

### Design

We used a 3 (image type: Caucasian faces vs. houses vs. words) × 2 (task: change detection vs. change localization) × 2 (image orientation: upright vs. inverted) design. Task, orientation, and image type were all manipulated within subjects. Task and image types were blocked while orientation was intermixed.

### Procedure

In the change localization condition, participants completed 60 trials for each type of material; each of six original faces, six original houses, and six original words was tested with each of five possible feature variations, once in an upright orientation and once upside down. Both images in each trial were displayed in the same orientation, upright or inverted. Inverted and upright images were randomly intermixed. Each material was preceded by training with 20 trials. In all trials, there was a change from the original image. Each trial presented an original image (1.5 s), a blank mask (0.3 s), and then a test image (1.5 s), followed by an instruction screen. In the change localization condition, the instruction screen read, “Please identify what change you believe could have occurred.” The five possibilities were listed, and participants used the keyboard (1 to 5 keys) to signal the chosen feature.

In the change detection condition, participants completed 120 trials for each type of material. In half of the trials, there was a change from the original image, while in the other half of the trials, there was no change. Each image was shown an equal number of times upright and inverted, and the inverted and upright images were randomly intermixed. Each trial presented an original image (1.5 s), a blank mask (0.3 s), and then a test image (1.5 s), followed by an instruction screen. In the change detection condition*,* the screen read, “Did a change occur in the face [house; word] you were originally presented?” Participants used the “yes” or “no” labeled keyboards to signal their options.

For both tasks, stimuli were presented on a 17″ cathode-ray tube monitor, and E-Prime 2.0 was used to control stimulus presentation and response recording. Participants were allowed to rest between blocks (a combination of tasks and materials). The experiment lasted for 45 or 50 min on average.

## Results

**Figure 2.**
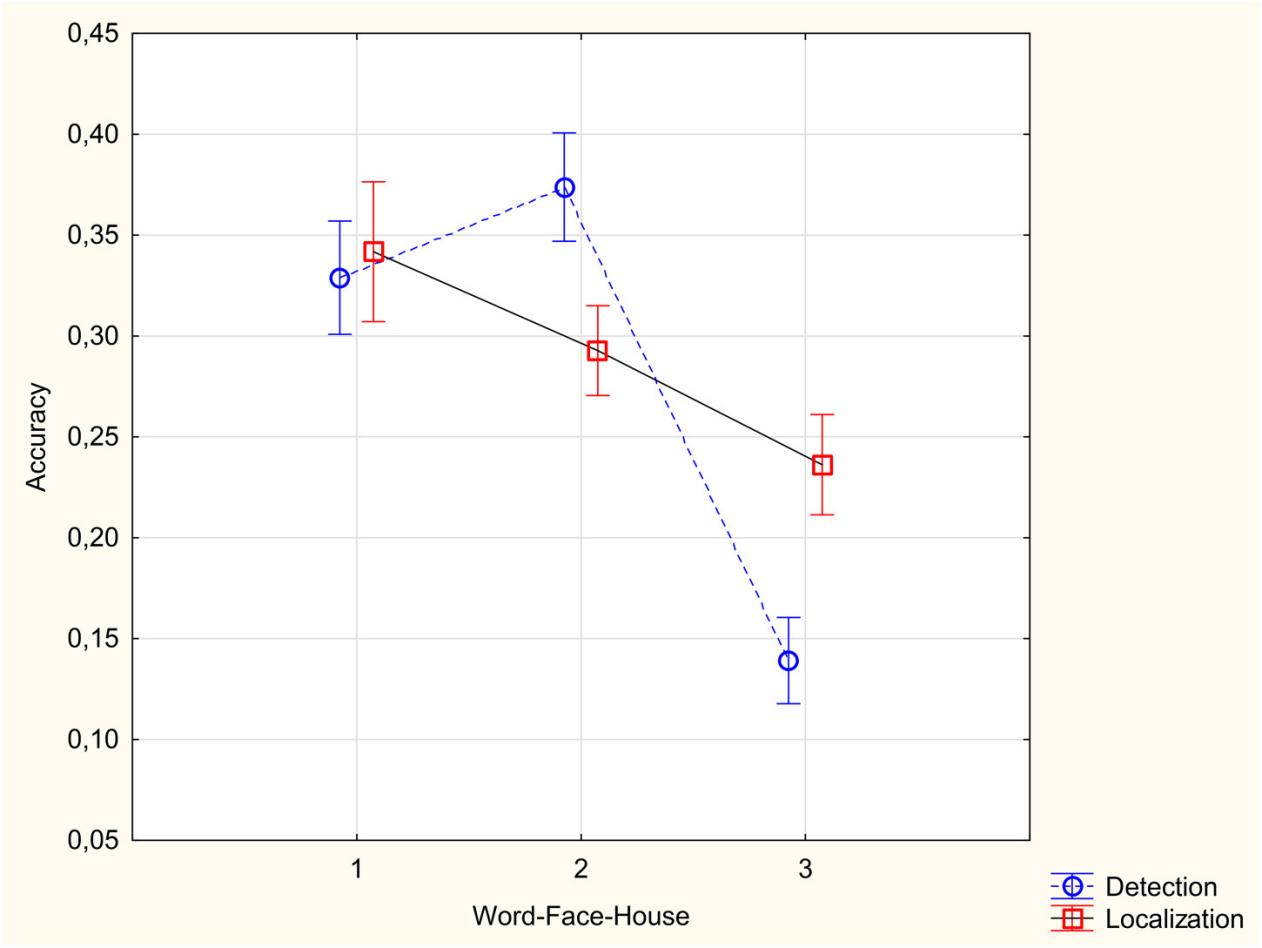
Accuracy in detection and localization tasks for different stimulus categories.

As the chance performance was different for the two tasks (50% for detection and 20% for localization), each participant's accuracy was corrected for chance using the formula (%correct − %chance)/(100% − %chance). Figure 2 shows the accuracy of detection and localization tasks for different object categories. Results presented in Figure 2 are the mean of the upright and inverted trials.

An ANOVA was conducted with 3 (image type: faces vs. houses vs. words) × 2 (task: change detection vs. change localization) × 2 (image orientation: upright vs. inverted). The most important analyses concern the interaction between task and category, and the three-way interaction. The category × task interaction was significant, *F*(2, 154) = 33.64, *p* < .0001, *η*_p_^2^^ ^= .30. Accuracy was higher for change detection than change localization only for faces, *t*(77)= 6.33, *p* < .0001 (Figure 2). In contrast, accuracy was higher in localization than detection for houses, *t*(77)= 7.32, *p* < .0001 (Figure 2). For words, change detection and change localization tasks did not differ in accuracy, *t*(77)= 0.65, *p* = .52 (Figure 2). The three-way interaction between category, task, and orientation was not significant, *F*(2, 154)= 1.8, *p *= .17 *η*_p_^2^^ ^= .02*.* Other significant effects include the main effect of category, *F*(2, 154) = 90.49, *p* < .0001, *η*_p_^2^^ ^= .54, with higher accuracy for faces and words than houses, the main effect of orientation, *F*(1, 71) = 85.52, *p* < .0001, *η*_p_^2^^ ^= .53, with higher accuracy for the upright condition, and the interaction between category and orientation, *F*(2, 154) = 44.24, *p* < .0001, *η*_p_^2^^ ^= .36, with higher accuracy for words and faces in the upright than upside down conditions, but no difference between the upright and upside down conditions for houses.

## Discussion

Our findings showed the relative ability to detect a change versus localize the change depending on the object category. Detection was easier than localization for faces, and localization was easier than detection for houses. This is expected as faces are processed holistically while houses are processed by parts. The data of Matis and Kahan (2014) and Poljac et al. (2012) add to this by showing that the detection benefit and identification impairment observed when faces are processed generalize to other globally processed stimuli. However, for words, which are also processed holistically (cf. the evidence reviewed in the Introduction), there was no clear difference in our study between detection and localization performance.

Traditionally, whereas it is deemed advantageous to represent distinct word parts or letters for word recognition, the ability to explicitly represent entire objects or words has been deemed relatively unnecessary (Farah et al., 1998). Words are considered unrecognizable unless their letters are individually distinguishable, indicating that words are identified by letter parts (Pelli et al., 2003). Recent research has shown, however, that expert word recognition necessitates attention to all parts. Applying the face composite paradigm to words in alphabetic (English and Portuguese) and nonalphabetic (Chinese) writing systems (Chen et al., 2013; Ventura et al., 2017; Wong, Bukach, Hsiao, et al., 2012; Wong, Bukach, Yuen, et al., 2011), it was observed that the irrelevant part of a word interfered with visual judgment despite observer's explicit attempts to ignore it, indicating that observers must pay attention to all parts of the word. This composite effect was larger for words in a person's native writing system and for words compared to nonwords, indicating that the holistic processing style is acquired through perceptual experience (Wong, Bukach, Yuen, et al., 2011).

The absence of a general change detection advantage over change localization for words, despite evidence that both faces and words are processed holistically, reflects most probably different processes for the holistic processing of faces and words with a cost of holistic processing observed only for faces, but not for words. Considering the ample evidence for holistic processing for words reviewed in the Introduction, this difference might mean that words are more resistant to the more “negative” effects of holistic processing, specifically for the change localization.

Being an object of visual expertise processed holistically, visual words are also a linguistic entity. In Ventura et al. (2019), the word composite effect was found for phonologically consistent words but not for phonologically inconsistent words. These findings suggest that the holistic processing of visual words is modulated by the automatic activation of lexical phonological representations. Being an object of visual expertise for which linguistic information is important, letter position information, is also crucial. Efficient visual word recognition (i.e., fast access to abstract orthographic representations at the mental lexicon, which then act as a key interface to phonological and conceptual representations) requires fast identification of letters (from a limited set) and of their position within-word (e.g., 〈GOD〉 is different than 〈COD〉, although they only differ in a minute horizontal segment of the first letter; and 〈GOD〉 is different than 〈DOG〉 . Orthographic processing or the processing of information about features/letter identities and letter positions is fundamental to the reading process (e.g., Grainger, 2017). Reading is both a visual and linguistic skill and orthographic processing occupies the key interface between visual and linguistic processing. In a nutshell, orthographic processing is here viewed as the “mid-level vision” of reading, providing the interface between lower-level visual processing and higher-level linguistic processing (e.g., Grainger, 2017). From this perspective, single-word reading is a combination of visual object identification processes and linguistic processing, with orthographic processing acting as the interface between the two. That is, orthographic processing allows generic visual processing mechanisms to contact the linguistic processing that is specific to word stimuli compared with other kinds of visual objects. Despite the possibility of some noise in encoding positional information (e.g., bigrams; Grainger & van Heuven, 2003; Perea & Lupker, 2003), the information about the location of features/letters is crucial in orthographic processing. This might rise performance in change localization and thus equalize performance in detection and localization tasks.

In conclusion, we found clear benefits of holistic processing for faces. For houses, change localization was easier. The difference between change localization and change detection was not obvious for words. There is a lesser cost of change localization for words, most probably due to the importance of localization of features/letters in word visual orthographic processing. Considering the cost of holistic processing there seems then to be a continuum from objects of expertise—faces to objects. Objects of expertise-words seem to be in the middle.
